# YOLO11-WLBS: an efficient model for pavement defect detection

**DOI:** 10.1038/s41598-026-35743-8

**Published:** 2026-01-15

**Authors:** Junqi Lin, Pinxin Wang, Yunkai Ruan, Yunqiang Sun

**Affiliations:** 1 College of Transportation and Civil Engineering, Fujian Agriculture and Forestry University, Fuzhou, 350002 China; 2 Zhejiang Zhe Jiao Testing Technology Co., Ltd., Hangzhou, 310000 China

**Keywords:** Pavement defect detection, YOLO11, Detection precision, Lightweight performance, Generalization, Engineering, Mathematics and computing

## Abstract

Pavement defects pose serious threats to traffic safety, pavement durability, and operational efficiency. To achieve accurate and real-time identification of pavement defects, this study proposes an enhanced lightweight model, YOLO11-WLBS, which integrates four improved modules—Wavelet Transform Convolution, Lightweight Adaptive Extraction, Bidirectional Feature Pyramid Network, and Simple Attention—into the YOLO11 framework. Each module’s contribution is verified through ablation experiments. The proposed model achieves a precision of 0.947, recall of 0.895, F1-score of 0.895, mAP@0.5 of 0.944, and mAP@0.5–0.95 of 0.703, demonstrating high accuracy and efficiency. Compared with the baseline YOLO11, YOLO11-WLBS improves precision by 6.4%, recall by 15.8%, and mAP@0.5 by 12.2%, while reducing parameters by 25.5%. The model maintains excellent detection performance under extreme lighting and blurring conditions and exhibits strong generalization in cross-dataset applications. These results indicate that YOLO11-WLBS provides an efficient and robust solution for intelligent pavement defect detection and offers practical potential for real-time deployment on edge devices in pavement maintenance and infrastructure monitoring systems.

## Introduction

As global pavement networks expand and traffic volumes increase, pavement wear and defects have intensified, leading to reduced structural integrity, lower driving comfort, and a higher risk of accidents^1^. Timely detection and reporting to relevant authorities enable early intervention, extending pavement lifespan and reducing accident risk. Therefore, detecting pavement defects is essential for maintenance and traffic safety, contributing to overall safety, transportation efficiency, and lower maintenance costs^2^. Therefore, efficient and accurate pavement defect detection is an important part of road maintenance and traffic safety management^3^.

Traditional detection methods rely on manual inspection or simple image processing techniques, which are inefficient and highly dependent on inspector expertise^4–6^. Human involvement and technological limitations cause these methods to perform poorly in both efficiency and accuracy^7^. As maintenance demands rise, these limitations become more pronounced. Thus, researchers have applied machine learning classifiers^8–10^ to enhance the automation and accuracy of pavement defect detection. However, these methods still rely on manually designed features, limiting their ability to handle complex backgrounds and diverse defects^11^. Given the limitations of manually designed features, researchers have increasingly turned to deep learning, which can automatically learn hierarchical features from raw images^12^. Multi-layer neural networks, a common deep learning architecture, extract complex features from large datasets, improving both detection efficiency and accuracy^13^. These models reduce reliance on manual intervention and handle diverse defect types under complex conditions^14^. Among deep learning architectures, Convolutional Neural Networks (CNNs) have become the preferred choice for image-based tasks, as their convolutional layers can effectively capture spatial and contextual features^15^.

Breakthroughs in CNNs have enabled more effective automatic pavement defect detection^16^. CNN-based detection algorithms such as You Only Look Once (YOLO)^17^, Faster R-CNN^18^, MASK R-CNN^19^, and Single Shot MultiBox Detector (SSD)^20^ have been widely applied, performing automatic defect detection with high accuracy. Target detection methods are generally categorized into two-stage approaches (such as Faster R-CNN and MASK R-CNN), which first generate candidate regions and then classify them, and single-stage approaches (such as YOLO and SSD), which simultaneously perform localization and classification. Among these, YOLO, as a representative one-stage algorithm, employs an end-to-end framework that efficiently extracts image features while balancing detection speed and accuracy^21^, making it particularly suitable for pavement defect detection^22^. Majidifard et al.^23^ used YOLOv2 and Faster R-CNN to classify pavement defects. In the experiment, the YOLOv2 model achieved an F1 score of 0.84, which proved its effectiveness in defect detection. Ma and Chen^24^ proposed an enhanced YOLOv5-based pavement defect detection model that integrates dual-branch channel and spatial attention mechanisms with GIoU loss, significantly improving small-object detection accuracy, localization precision, and overall performance in pavement condition evaluation. Zhang et al.^25^ proposed an improved YOLOv8-based model, SMG-YOLOv8, which integrates spatial-to-depth and multi-scale attention modules. This model maintains excellent detection performance in asphalt pavement distress detection across various scenarios while reducing the number of model parameters. Li et al.^26^ proposed a road surface crack detection model based on lightweight deformable convolution, YOLO-DGVG, which enhances the model’s adaptability to crack shapes, reduces parameters by 22%, and improves recall and mAP by 1.6%, demonstrating strong performance and making it suitable for edge deployment.

Most existing on YOLO-based pavement defect detection have largely revolved around a common challenge—how to achieve an optimal balance between detection accuracy and efficiency^27^. Detection accuracy is often constrained by the intrinsic complexity of pavement defects, including irregular shapes, diverse textures, and multi-scale characteristics, as well as environmental interferences such as illumination variation and background noise^28^. On the other hand, efficiency is limited by the increasing depth and parameter scale of detection networks, which impose higher computational demands and hinder real-time deployment^29^. Therefore, designing a model that maintains high accuracy while ensuring computational efficiency has become a crucial direction for advancing pavement defect detection toward practical applications. To this end, we propose YOLO11-WLBS, an enhanced model based on YOLO11. The model is optimized across multiple dimensions for pavement defect detection, integrating Wavelet Transform Convolution (WTConv) for high-frequency feature enhancement, a Lightweight Adaptive Extraction module (LAE) for parameter reduction, a Bidirectional Feature Pyramid Network (BiFPN) for multi-scale feature fusion, and a Simple Attention Mechanism (SAM) for optimized feature weighting. These improvements collectively achieve a well-balanced enhancement in detection accuracy and computational efficiency.

## Methods

In order to balance detection accuracy and real-time performance for pavement defect detection, this paper proposes an enhanced YOLO11 architecture, named YOLO11-WLBS. The subsequent chapters will explain the architecture and its construction method in detail.

### Model architecture

**Fig. 1:**
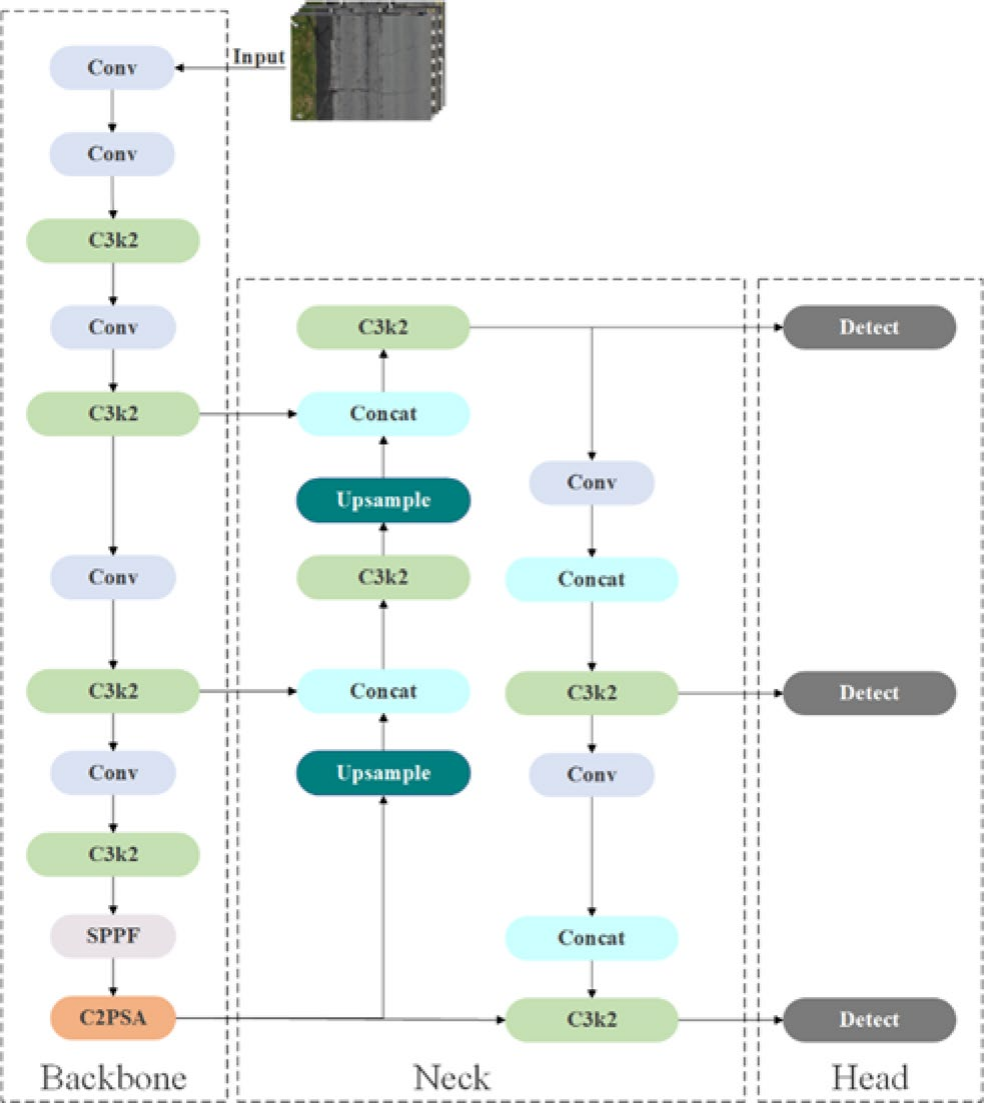
Architectures of the YOLO11.

In this study, we use YOLO11, the latest iteration of the YOLO series released by Ultralytics in 2024 (https://docs.ultralytics.com/models/yolo11), as the baseline model for the detection and identification of pavement defects. The overall architecture of YOLO11 is illustrated in Fig. 1. The YOLO11 retaining the conventional three-stage design comprising the Backbone, Neck, and Head (Fig. 1). The Backbone extracts feature from the input image through stacked convolutional layers, progressively capturing spatial and semantic information at multiple levels (Fig. 1). The Neck fuses and enhances multi-scale features to strengthen the representation of target objects (Fig. 1). The Head performs object classification and bounding box regression, yielding stable and highly accurate detection results (Fig. 1).

**Fig. 2:**
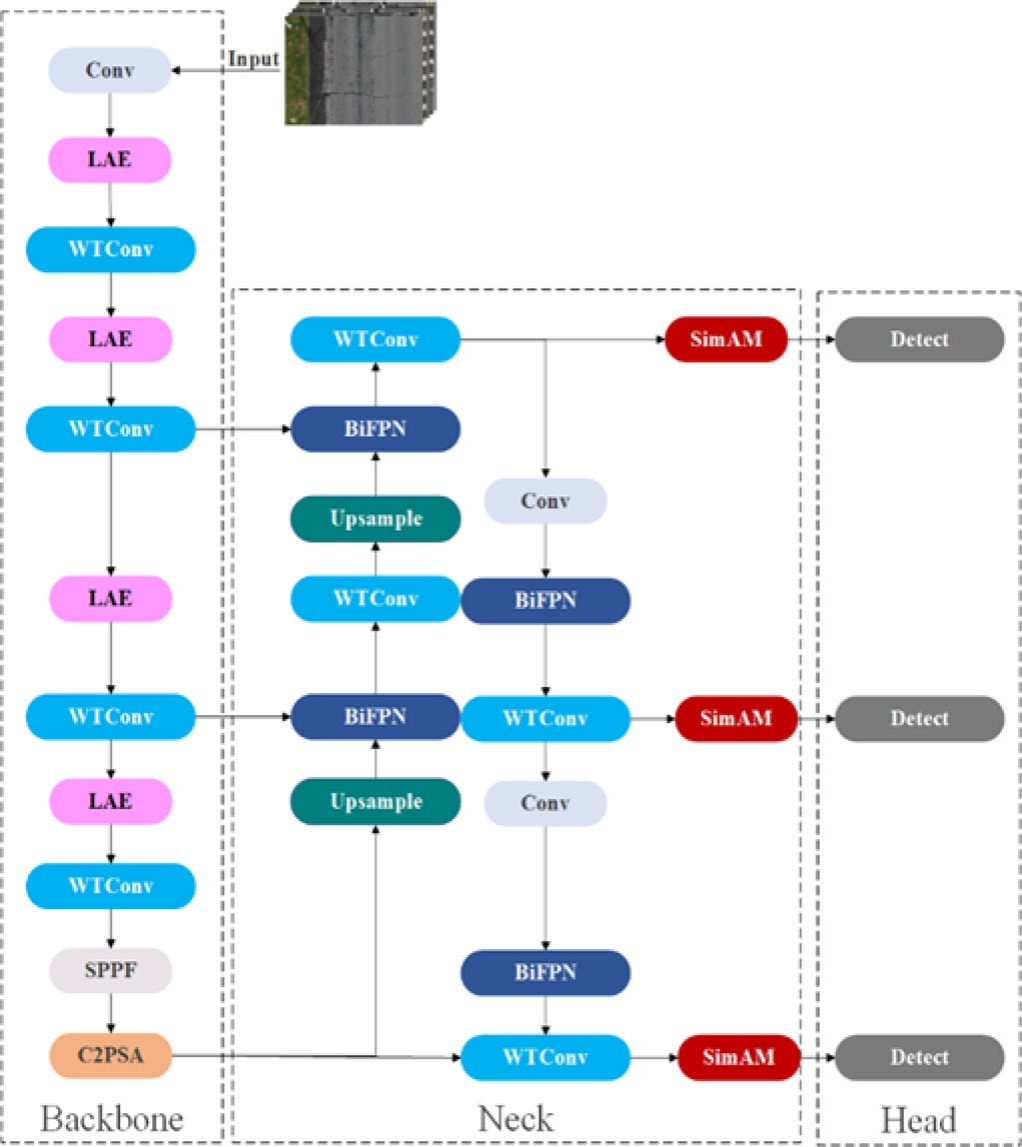
Architectures of the YOLO11-WLBS.

Although YOLO11 has demonstrated excellent performance in general object detections^30^, it may still exhibit limitations in pavement defect detection. For example, pavement defects often present low contrast, subtle textures, and irregular morphologies, which challenge YOLO11’s standard convolutional architecture in fully extracting high-frequency edge details and low-contrast texture features; small cracks or superficial defects may be missed or falsely detected. Thus, to achieve higher detection accuracy that aligns with the practical needs of pavement maintenance, this study proposed an enhanced model called YOLO11-WLBS (as shown in Fig. 2, this model is based on YOLO11 and integrates WTConv, LAE, BiFPN, and SAM modules).

YOLO11-WLBS, as a single-stage detector based on anchor boxes (YOLO11), can simultaneously process multiple defect types in a single road surface image^17^. The model generates multiple prediction boxes, each corresponding to a different defect category. Its head simultaneously calculates the category probability and confidence for each prediction box, enabling the network to independently classify and locate each defect^31^. The loss function comprehensively considers bounding box regression, category prediction, and target confidence, thereby learning the spatial location and category of multiple defects in the image during training, achieving efficient multi-defect recognition^32^. Next, we will provide a detailed introduction to the imported module.

### Wavelet transform convolution module

In image processing, information is generally categorized into low-frequency and high-frequency components^33^. Specifically, low-frequency information corresponds to smooth or large uniform regions, where pixel values change gradually. In contrast, high-frequency information represents edges, textures, and fine details, where pixel values vary sharply^34^. As a general-purpose object detection model, YOLO11 is designed to balance high- and low-frequency information, which is effective for typical object detections^17^. However, pavement defects are often small and characterized by pronounced high-frequency features^3^. YOLO11’s frequency-balancing strategy may limit its ability to capture pavement defects^35^.

**Fig. 3:**
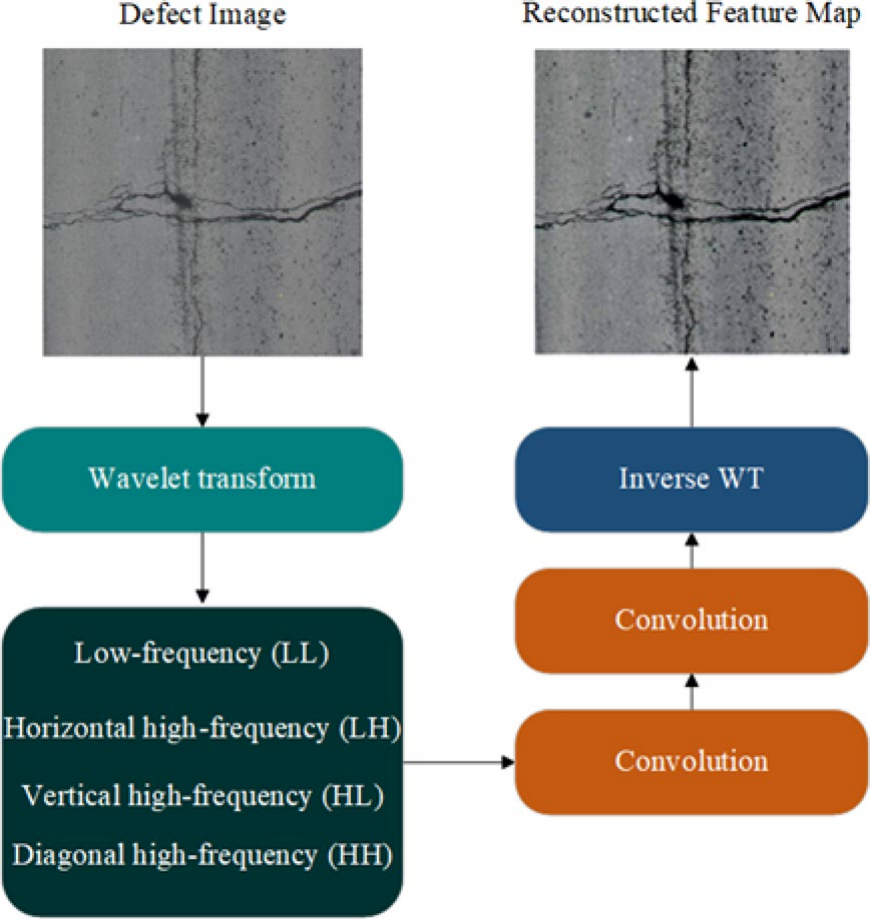
Schematic of the WTConv Module.

To this end, this study introduces the WTConv module. WTConv employs wavelet and inverse wavelet transforms to enhance the representation of high-frequency features (see Fig. 3 for the module structure). We use Haar wavelets (Daubechies-1)^36^ for wavelet decomposition and reconstruction. This is because the piecewise constant basis function of Haar wavelets renders it highly sensitive to intensity discontinuities and edge variations, thus enabling effective characterization of pavement defects dominated by high-frequency information such as cracks and pothole boundaries. Meanwhile, its concise orthogonal structure and compact support set lead to extremely low computational complexity in multi-scale decomposition, which is conducive to preserving the overall lightweight property of the model. Specifically, the input feature map is first decomposed into four sub-bands: low-frequency (*LL*), horizontal high-frequency (*LH*), vertical high-frequency (*HL*), and diagonal high-frequency (*HH*)^37^. Each sub-band is then processed with two basic convolution kernels (Formula 1), improving the representation of different frequency components, particularly enhancing sensitivity to small or irregular defects such as crack edges and fine textures. Subsequently, the module re-integrates the four sub-images through inverse wavelet transform (Formula 2) and restores them to a feature map consistent with the original input structure. Finally, the feature map, containing enhanced multi-frequency information reconstruction, is passed as input to the next layer of the model for subsequent feature extraction.

1 $${X^\prime}{\text{ }}={\text{ WTConv(}}X{\text{) = }}X^*{W_{dec}}$$

2 $${X^{\prime\prime}}={\mathrm{IWTConv(}}{X^\prime}{\text{) = }}\sum\limits_{{i \in \left\{ {LL,HH,LH,HL} \right\}}} {{X_i}^*W_{{rec}}^{i}}$$

where *X* is the input image, ${X^\prime}$ is the feature map after wavelet transformation, and ${X^{''}}$ is the original image structure after inverse wavelet transform. All of them have the same dimension ${{\mathbb{R}}^{C \times H \times W}}$ with *C* channels, height *H*, and width *W*; * is the 2D convolution operation; ${W_{dec}}$ is the wavelet decomposition filter bank used to extract four frequency sub-bands (*LL*,* LH*,* HL*, and *HH*.); ${X_i}$ is the components of each frequency sub-graph, and $W_{{rec}}^{i}$ is the corresponding reconstruction filter.

### Lightweight adaptive extraction module

**Fig. 4:**
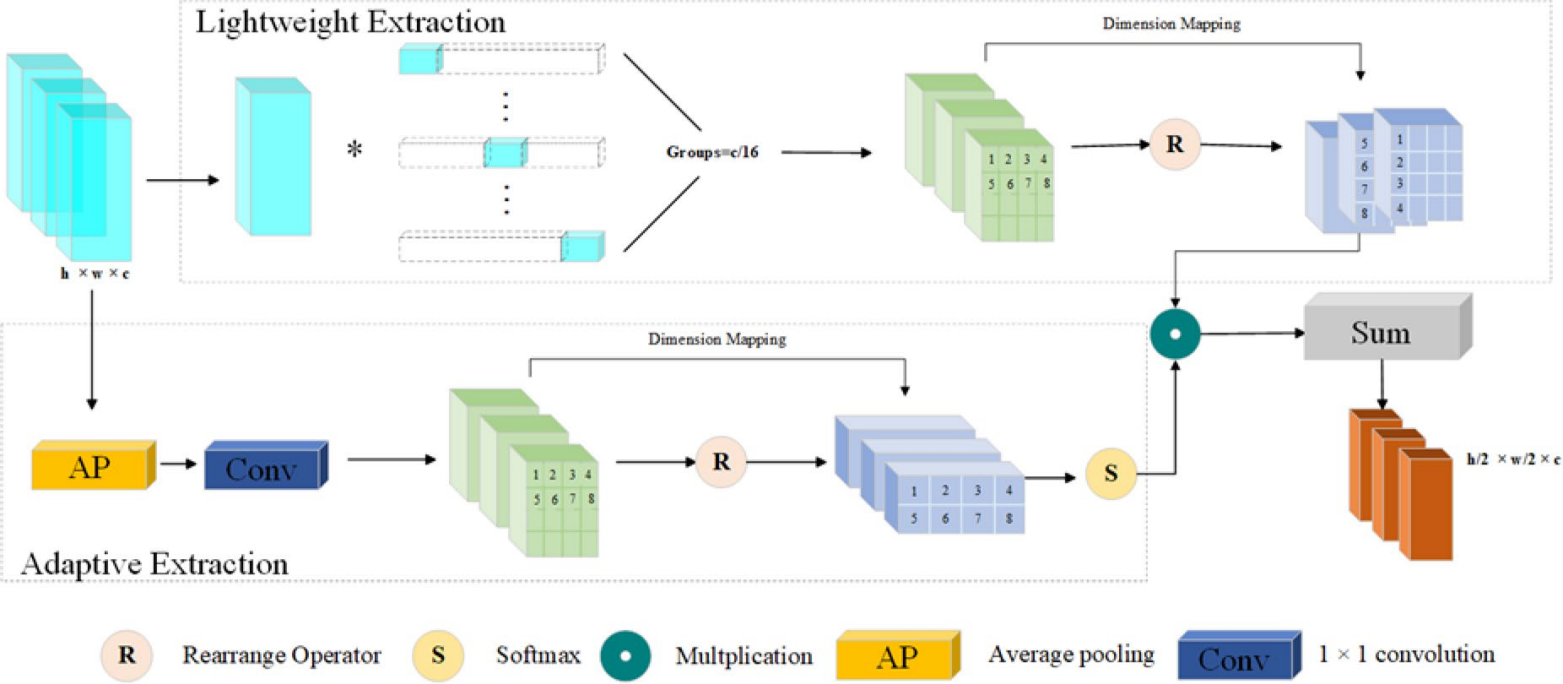
Schematic of the lightweight adaptive extraction module framework.

To enhance pavement defect-related feature extraction while maintaining computational efficiency, we integrate the LAE module (see Fig. 4 for the module structure) immediately after the convolutional Backbone.

The LAE module combines two main functions, lightweight extraction and adaptive extraction. Lightweight extraction employs grouped downsampling convolutions to obtain local spatial features (Formula 3). This operation uses a *3 × 3* convolution kernel with a stride of *2* and applies group convolution (*Groups = C/16*), dividing the channels into *C/16* subgroups for independent convolution within each group, thus effectively reducing parameters and computational cost. Adaptive extraction captures global semantic information via global average pooling followed by *1 × 1* convolutions, generating selective channel attention weights (Formula 4) to recalibrate features adaptively across channels. Finally, the local features and adaptive weights are fused element-wise (Formula 5), highlighting disease-sensitive regions and enhancing semantic expressiveness while maintaining a lightweight structure. The final output feature map has dimensions *H/2×W/2×C*, providing an efficient feature representation with reduced spatial resolution and enriched expressive power.

3 $${X_{Lightweight{\text{ }}Extraction}}{\text{ }}={\text{ Con}}{{\mathrm{v}}_{k=3,s=2,g=C/16}}{\mathrm{(}}X{\text{) }}$$

4 $$A{\text{ }}={\text{ }}Soft\hbox{max} (Con{v_{1 \times 1}}(Avgpool(X)){\text{ }}$$

5 $$Y{\text{ }}={\text{ }}{{\mathrm{X}}_{Lightweight{\text{ }}Extraction}} \odot A$$

where $X \in {{\mathbb{R}}^{C \times H \times W}}$ represents the input feature map, which has *C* channels, height *H*, and width W; ${X_{Lightweight{\text{ }}Extraction}} \in {{\mathbb{R}}^{C \times H/2 \times W/2}}$ represents the local feature map obtained through lightweight extraction. $A \in {{\mathbb{R}}^{C \times 1 \times 1}}$ is an adaptive channel attention weight generated by global average pooling and *1 × 1* convolution, and normalized by the *softmax* function. The average pooling and *softmax* operations are used only in adaptive extraction function, so their definitions remain consistent throughout the LAE module; $Y \in {{\mathbb{R}}^{C \times H/2 \times W/2}}$ is the final output feature map, which is obtained by fusing the locally extracted features *X*_*Lightweight Extraction*_ with the adaptive weights *A* by element-wise multiplication $\odot$.

### Bidirectional feature pyramid network module

**Fig. 5:**
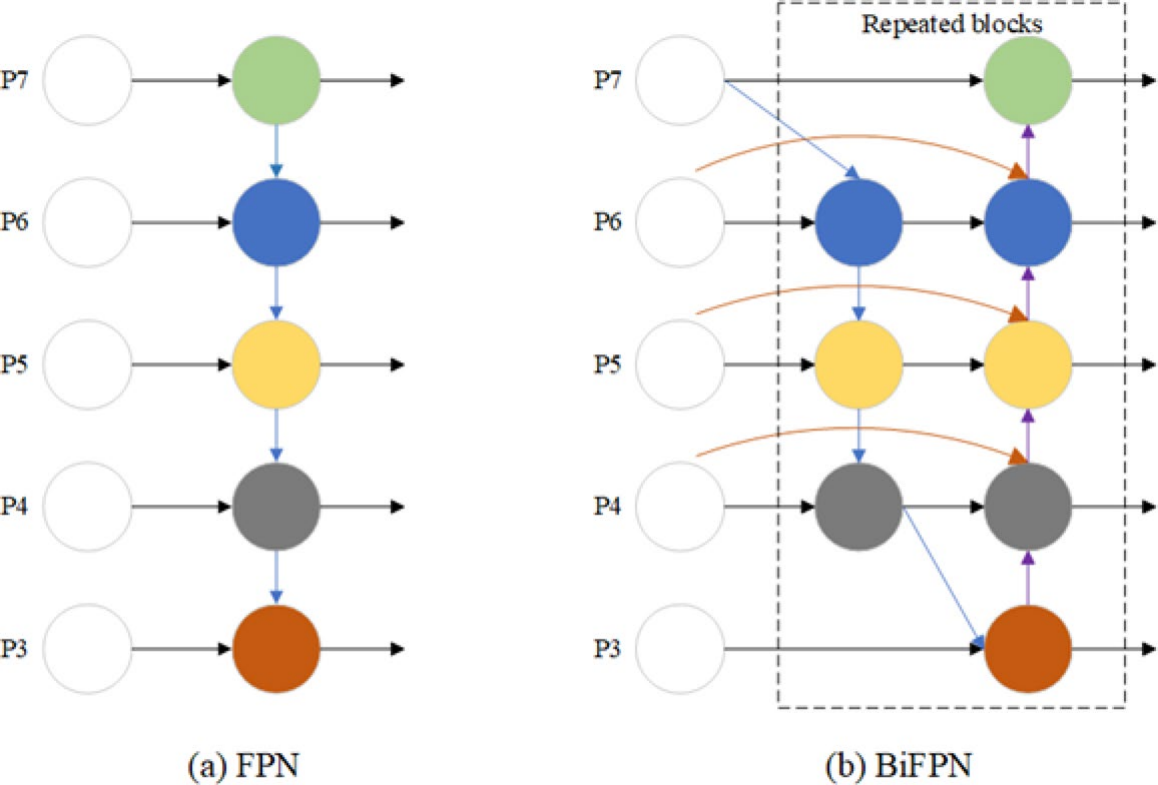
**(a)** Depicts the feature pyramid network, and **(b)** Depicts the bidirectional feature pyramid network.

The Neck of YOLO11 uses a Feature Pyramid Network (FPN) for multi-scale feature fusion. The FPN integrates high- and low-level features through a bottom-up pathway and lateral connections (Fig. 5(a)), enabling feature maps to balance semantic context and fine-grained details, with its direct fusion strategy reducing computational overhead and model complexity^38^. However, the one-way fusion mechanism may constrain the network’s ability to capture complex object morphologies, particularly for small or irregular pavement defects with intricate edge and texture patterns^39^. Here we incorporate the BiFPN in the Neck part (Fig. 5(b)). BiFPN enables efficient multi-level feature fusion through bidirectional information flow and learnable fusion weights, substantially enhancing the extraction of detailed features while preserving semantic integrity^40^. This bidirectional design effectively mitigates the semantic degradation that can result from the one-way transmission in FPN, thereby may improve the model’s capacity to comprehensively capture pavement defects.

The BiFPN module receives a set of multi-scale feature maps *P* (defined in formula 6.) from the backbone or upsampling/downsampling branches. Each input feature map participates in both top-down and bottom-up information transfer, achieving bidirectional multi-scale feature fusion. During training, each input feature map is batch normalized (Formula 7) and weighted by learnable fusion weights *w*_*ij*_ to generate intermediate output feature maps (Formula 8). This operation not only stabilizes the training process but also adaptively adjusts the importance of different input feature maps according to the fusion weights. Finally, combined with the upsampling/downsampling strategy, the normalized intermediate features are restored to their original scale and distribution to obtain the final output feature map *O*_*i*_ (Formula 9).

6 $$P{\text{ }}={\text{ }}\left\{ {{P_1},{P_2},{P_3}...,{P_n}} \right\}{\text{ , }}{P_j} \in {{\mathbb{R}}^{C \times H \times W}}$$

7 $${\hat {P}_j}{\text{ }}={\text{ }}\frac{{{P_j} - {\mu _j}}}{{{\sigma _j}}}$$

8 $${\hat {O}_i}{\text{ }}={\text{ }}\sum\limits_{{j=1}}^{n} {{w_{ij}} \cdot {{\hat {P}}_j}}$$

9 $${O_i}{\text{ }}={\text{ }}{\hat {O}_i} \cdot {\sigma _i}+{\mu _i}$$

where ${P_j}$ represents the *j-th* multi-scale feature map; ${\mu _j}$ and ${\sigma _j}$ are the mean and standard deviation of feature map; ${\hat {P}_j}$ is the normalized feature map; ${w_{ij}}$ is a learnable fusion weight used to adjust the contribution of each input feature map to the *i-th* output feature map; ${\hat {O}_i}$ is the intermediate output feature map after weighted fusion; ${\sigma _i}$ and ${\mu _i}$ are the standard deviation and mean used to restore the scale; ${O_i}$ is the final output feature map.

### Simple attention module

To enhance the model’s capacity for characterizing pavement defects in complex, multi-scale contexts, this study incorporates the SAM into the Neck component. The SAM is a lightweight attention mechanism that identifies key features by quantifying information differences between neurons^41^. By emphasizing salient regions without relying on computationally intensive matrix operations, this mechanism effectively improves the detection of small cracks and low-contrast defects. The SAM module measures the importance of each neuron *x*_*i*_ in the feature map *X* from the previous layer by calculating its energy *E*_*i*_. Energy scale differences are eliminated by normalizing *E*_*i*_ (Formula 10), and the final weighted features are generated using the sigmoid activation function (Formula 11) to highlight key feature regions.

10 $${E_i}=\frac{1}{{2n}}\sum\limits_{j}^{{}} {{{({x_i} - {x_j})}^2}++\lambda {w_{}}^{2}}$$

11 $${\hat {x}_i}=sigmoid(\frac{{{E_i} - {\mu _E}}}{{{\sigma _E}}})$$

where ${x_i}$ is the feature value of the $i - th$ neuron; ${x_j}$ represents the feature values of other neurons in the feature map; *n* is the number of channels in the feature map; *w* is a learnable parameter; $\lambda$ is the regularization coefficient to prevent overfitting; ${\hat {x}_i}$ represents the output feature of the *i-th* neuron after attention weighting; ${\mu _E}$ and ${\sigma _E}$ are the mean and standard deviation of the energy *E*.

## Experimental setup an evaluation metrics

### Datasets

**Fig. 6:**
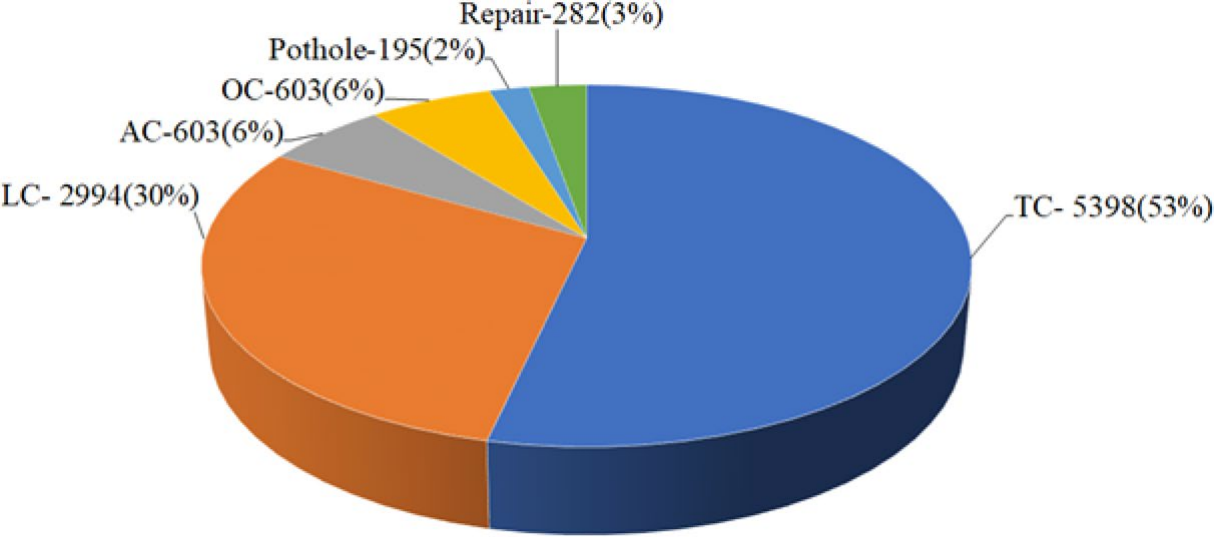
Distribution of pavement defects types in the dataset.

The dataset used in this study is UAV-PDD2023 (https://zenodo.org/records/8429208), comprising 2,440 high-resolution road surface images (2592 × 1944) taken by drones, clearly showing even tiny cracks and surface defects. The dataset features favorable lighting conditions and diverse scene environments, providing rich features for model learning. This dataset includes six types of pavement defect: transverse cracks (TC), longitudinal cracks (LC), alligator cracks (AC), oblique cracks (OC), potholes, and repairs (Fig. 6). A total of 10,075 pavement defect instances were annotated. Each defect is independently annotated with a bounding box and assigned a category label. A single image typically contains multiple defect types, with each defect corresponding to an independent anchor box, enabling the network to learn the spatial location and category of each defect. Furthermore, the dataset contains real-world defect co-occurrences and occasional overlaps, which helps the model master the ability to recognize complex multi-defect scenes during training. The dataset is then divided into training, validation, and test sets at a ratio of 7:2:1. This division method has been widely used in the field of deep learning and can achieve a good balance between model learning ability and generalization performance^42^.

### Experimental environment and parameter settings

All model training in this study was conducted on a personal workstation. It is equipped with two Intel Xeon 6226R CPUs, one NVIDIA GeForce RTX 4080 Super GPU, and 256 GB of RAM (Table 1). The software environment for the model development is based on the Windows10 operating system. The model operation is built on the Python 3.9.18 environment. The core dependent libraries include PyTorch 2.0.0, CUDA 11.8, Torchvision 0.15.1, OpenCV 4.11.0, and Ultralytics 8.3.3 (Table 1).

**Table 1:** Experimental environment.

Hardware	CPU	Inter(R) Xeon(R) Gold 6226R
	GPU	NVIDIA GeForce RTX 4080 Super
Software	OS	Windows10
	Python	3.9.18
	PyTorc	2.0.0
	CUDA	11.8
	Torchvision	0.15.1
	OpenCV	4.11.0
	Ultralytics	8.3.3

Table 2 details the key training parameters, including batch size, optimizer type, learning rate, training epochs, patient epochs, number of workers, input size, and random seed. Data augmentation is performed using Mosaic stitching. The learning rate uses a Cosine Annealing decay strategy. Three warm-up epochs were included before training, employing linear warm-up to gradually increase the learning rate from 0 to an initial learning rate of 0.01. For the SGD optimizer, the hyperparameters were set as momentum = 0.937 and weight decay = 0.0005. During the training process, automatic mixed precision was used to improve memory utilization and accelerate the training efficiency. To obtain more stable model weights, we enabled the exponential moving average method, smoothly averaging historical and recent training updates. The confidence threshold and non-maximum suppression threshold adopted the default values of the YOLO framework, which are 0.25 and 0.45, respectively. All subsequent experiments used the same parameter and mechanism settings to ensure fairness in performance comparisons among all models. Other parameters not explicitly specified used the default settings for the YOLO11 model.

**Table 2:** Parameter settings.

Parameters Setting	Batch size	64
	Optimizer	SGD
	learning rate	0.01
	epochs	300
	Patience epochs	100
	Number of workers	16
	Input size	640 × 640
	Random seed	42

### Model evaluation metrics

This study evaluates the model’s applicability to pavement defect detection using metrics consistent with the YOLO series: Precision (P), Recall (R), F1-Score (F1), and Mean Average Precision (mAP). Specifically, mAP includes mAP@0.5, which measures the proportion of predictions with an Intersection over Union (IoU) of at least 0.5, and mAP@0.50–0.95, which averages mAP across IoU thresholds from 0.5 to 0.95. The corresponding formulas are provided in formula 12–17^43^. During the inference phase, the model generates multiple prediction boxes for each image, with each box independently outputting a class probability and confidence score. Performance metrics (Precision, Recall, F1-Score, mAP@0.5, and mAP@0.50–0.95) are calculated based on all predicted defects, thus comprehensively evaluating the model’s detection capability in multi-defect scenarios.

P refers to the proportion of true positive samples among all samples predicted as positive by the model:

12 $$P=\frac{{TP}}{{TP+FP}}$$

where TP (True Positive) represents the number of samples predicted as positive and actually positive; FP (False Positive) represents the number of samples predicted as positive but actually negative.

R refers to the proportion of true positive instances correctly predicted by the model, relative to all actual positive instances:

13 $$R=\frac{{TP}}{{TP+FN}}$$

where FN (False Negatives) represents the number of samples predicted to be negative but actually positive.

F1 is the harmonic mean of P and R, serving as a comprehensive measure that balances both metrics.

14 $$F1=2 \times \frac{{P \times R}}{{P+R}}$$

mAP is a widely used metric for evaluating model performance in multi-class detection tasks. It represents the mean of Average Precision (AP) across all categories, with AP being calculated from the P-R curve at various IoU thresholds. For each category, AP is determined by computing precision and recall at different IoU thresholds, plotting the P-R curve, and then calculating the area under the curve. The formula for AP is as follow:

15 $$AP=\int_{0}^{1} {P(r)dr}$$

where $P(r)$ represents the precision under a certain recall rate *r*.

mAP@0.5 represents the mAP when the IoU is greater than or equal to 0.5. Specifically, for each category, a prediction is considered correct only when the IoU between the predicted and ground truth boxes meets or exceeds 0.5. The formula is:

16 $$mAP@0.5=\frac{1}{N}\sum\limits_{{i=1}}^{N} {A{P_i}(0.5)}$$

where *N* is the number of categories and $A{P_i}(0.5)$ is the AP of the $i - th$ category when $IoU{\text{ }}>={\text{ }}0.5$.

mAP@0.5–0.95 is the average mAP calculated under multiple IoU thresholds (from 0.5 to 0.95, increasing by 0.05 each time) for each category. The formula is:

17 $$mAP@0.5 - 0.95=\frac{1}{N}\sum\limits_{{i=1}}^{N} {\frac{1}{{10}}\sum\limits_{{j=1}}^{{10}} {A{P_i}(Io{U_j})} }$$

where $Io{U_j}=(0.5+0.05 \times (j - 1))$ indicates the IoU threshold at step $A{P_i}(Io{U_j})$; represents the Average Precision of category *i* at the IoU threshold $Io{U_j}$.

## Results

### Baseline model

Pavement defect detection presents significant challenges: cracks exhibit diverse morphologies, small sizes, and complex backgrounds, demanding high accuracy and robustness from detection models. To systematically evaluate the suitability of various object detection models for pavement defect detection, this study conducted comparative experiments. To ensure comparability between models, all baseline models used the same confidence threshold (0.25) and non-maximum suppression threshold (0.45), and the same IoU matching rule was used uniformly when calculating all evaluation metrics. All evaluation metrics reported in the subsequent tables are calculated exclusively on the test set to ensure a fair and unbiased performance assessment and to avoid any potential data leakage from the training process.

The experiments first examined classic object detection models, including the one-stage SSD^20^, and the two-stage faster R-CNN^44^ and mask R-CNN^19^. The results show that the detection accuracy of SSD model is relatively poor, with a precision of 0.572, a recall of 0.388, and a F1 of 0.462, moreover, the parameter count reaches 32.77 M (Table 3). The faster R-CNN and mask R-CNN models exhibit improved precision compared to the SSD model, with values of 0.636 and 0.659 respectively (Table 3); however, their overall accuracy remains relatively low (Table 3). The YOLO algorithm is an essential object detection method, enabling direct end-to-end image detection with wide applications. We further compared the detection results of different YOLO series, including YOLOv5^45^, YOLOv8^46^, YOLOv9^47^, YOLOv10^48^, and YOLO11^49^. The results indicate that there are differences in detection accuracy among the various YOLO models; however, their accuracy exhibits a significant improvement compared to the SSD, faster R-CNN and mask R-CNN models (Table 3). Among these models, the detection precision of YOLOv5 is 0.763, while the precision of the other models all exceeds 0.8 (Table 3). We note that with the iteration of YOLO versions, the detection accuracy has also improved; among them, YOLO11 attains the highest accuracy, with a precision of 0.853, a recall of 0.698, a F1 of 0.768, an mAP@0.50 of 0.797, and an mAP@0.50–0.95 of 0.624, while maintaining a moderate parameter count of 20.06 M (Table 3).

To investigate the effects of network depth, width, and overall complexity on detection performance and computational efficiency, we then conducted extensive experimental comparisons under different YOLO variants: nano, small, medium, large, and extra large (https://docs.ultralytics.com/zh/models/yolo11/). Table 4 shows the results of five variants of YOLO11, namely YOLO11n, YOLO11s, YOLO11m, YOLO11l, and YOLO11x (https://docs.ultralytics.com/zh/models/yolo11/). YOLO11n is a lightweight variant, offering fast inference speed and a low parameter count. YOLO11s and YOLO11m correspond to small and medium variants, respectively, achieving a balance between computational efficiency and detection accuracy. YOLO11l and YOLO11x are larger variants aimed at improving accuracy but at substantially higher computational cost^50–52^. The results indicate that YOLO11n and YOLO11s exhibits a relatively low detection precision, with values of 0.683 and 0.763, respectively (Table 4). The detection precision of YOLO11m, YOLO11l, and YOLO11x are relatively close, with value of 0.853, 0.854, and 0.841, respectively (Table 4). We found that although YOLO11l achieves the highest detection precision, its parameter count reaches 25.32 M (Table 4). In contrast, YOLO11m, while its detection precision is 0.01 lower than that of YOLO11l, reduces the parameter count to 20.06 M (Table 4). Considering both detection performance and computational efficiency, YOLO11m was selected as the baseline model for subsequent analysis.

Though compared with existing models, YOLO11m demonstrates the highest detection precision for pavement defects (Table 3), its overall detection precision (0.853) indicates that further improvements are feasible. Therefore, this study introduces targeted modules, including the WTConv, BiFPN, LAE, and SAM, into the YOLO11 model (Fig. 2). Ablation experiments are conducted to quantify the contribution of each module to overall performance, providing guidance for further model optimization.

**Table 3:** Performance comparison of object detection models.

Models	*P*	*R*	F_1_	mAP@0.50	mAP@0.50–0.95	Para/M
SSD	0.572	0.388	0.462	0.531	0.332	32.77
Faster R-CNN	0.636	0.451	0.528	0.598	0.391	26.21
Mask R-CNN	0.659	0.486	0.560	0.611	0.403	25.32
YOLOv5	0.763	0.524	0.621	0.731	0.549	22.18
YOLOv8	0.823	0.624	0.714	0.775	0.619	23.27
YOLOv9	0.832	0.625	0.714	0.765	0.613	16.78
YOLOv10	0.831	0.632	0.718	0.769	0.596	16.58
YOLO11	0.853	0.698	0.768	0.797	0.624	20.06

**Table 4:** Performance of YOLO11 variant models.

Models	*P*	*R*	F_1_	mAP@0.50	mAP@0.50–0.95	Para/M
YOLO11n	0.683	0.588	0.632	0.601	0.453	2.59
YOLO11s	0.763	0.600	0.672	0.692	0.462	9.43
YOLO11m	0.853	0.698	0.768	0.797	0.624	20.06
YOLO11l	0.854	0.707	0.775	0.802	0.625	25.32
YOLO11x	0.841	0.652	0.735	0.793	0.618	56.88

### Ablation experiments

After validating the effectiveness of YOLO11m as a reliable baseline model, this study introduces four targeted modules to address its limitations in pavement defect detection: the WTConv for enhanced high-frequency texture feature extraction, the LAE for reduced model complexity and enhanced deployment efficiency while maintaining detection precision, the BiFPN for optimized cross-scale feature fusion, and the SAM for improved attention allocation. To verify the rationality and necessity of these structural improvements, this section conducts systematic ablation. The experimental sequence was arranged according to the hierarchical position of each module in the target detection network and its role in the feature processing flow^53^, in order to quantitatively analyze the individual contributions and synergistic effects of each module in pavement defect detection.

**Table 5:** Ablation experiments.

Baseline	WTConv	LAE	BiFPN	SAM	*P*	*R*	F1	mAP@0.5	mAP@0.50–0.95	Para/M
YOLO11m					0.853	0.698	0.768	0.797	0.624	20.06
	√				0.875	0.720	0.790	0.830	0.663	22.31
		√			0.859	0.711	0.778	0.813	0.631	13.92
			√		0.881	0.726	0.796	0.828	0.655	21.64
				√	0.867	0.712	0.781	0.831	0.638	20.07
	√	√			0.909	0.754	0.824	0.883	0.675	14.25
	√		√		0.911	0.756	0.826	0.881	0.677	25.28
	√			√	0.902	0.748	0.818	0.876	0.665	24.28
		√	√		0.896	0.741	0.811	0.866	0.658	14.13
		√		√	0.893	0.738	0.809	0.863	0.636	14.10
			√	√	0.887	0.735	0.804	0.860	0.643	21.65
	√	√	√		0.916	0.814	0.863	0.891	0.685	14.83
		√	√	√	0.918	0.824	0.866	0.896	0.688	14.57
	√	√		√	0.919	0.821	0.867	0.892	0.678	14.61
	√		√	√	0.920	0.818	0.868	0.897	0.692	25.29
	√	√	√	√	0.947	0.895	0.920	0.944	0.703	14.94

The results show that compared with the baseline model, the detection accuracy of the model is improved after adding each individual module (Table 5), which proves the effectiveness of the four modules. Among them, the baseline model enhanced with WTConv achieves a precision improvement of 0.022, a recall improvement of 0.022, a F1 improvement of 0.022, an mAP@0.5 improvement of 0.033, and an mAP@0.5–0.95 improvement of 0.039 (Table 5). The model enhanced with LAE increases the precision, recall, F1, mAP@0.5, and mAP@0.5–0.95 by 0.006, 0.013, 0.01, 0.016, and 0.007 respectively. Simultaneously, the number of parameters decreases by 6.14 M. (Table 5). The BiFPN-enhanced model achieves improvements of 0.028, 0.028, 0.028, 0.031, and 0.031 in precision, recall, F1, mAP@0.5, and mAP@0.5–0.95, respectively (Table 5). When integrated with SAM, the model shows respective increases of 0.014, 0.014, 0.013, 0.034, and 0.014 in precision, recall, F1, mAP@0.5 and mAP@0.5–0.95 (Table 5). It can be seen that when each individual module is added, the model enhanced with BiFPN achieves the highest accuracy improvement, followed by WTConv (Table 5), which confirms the critical roles of BiFPN in multi-scale feature fusion and WTConv in high-frequency feature extraction. Although the SAM provides relatively modest improvements, it significantly enhances the model’s capability to focus on pavement defects within complex backgrounds under high-threshold conditions (mAP@0.50–0.95 increases by 0.031 compared to the baseline model). The LAE effectively reduces the number of parameters (by approximately 30.63% compared to the baseline model) while maintaining accuracy, thereby improving computational efficiency and facilitating mobile deployment.

Experiments that integrated multiple modules further demonstrated the complementarity between each component (Table 5). When integrating only two modules, the model combining WTConv with BiFPN yielded significant gains of accuracy; and the subsequent addition of SAM further improved performance (Table 5). Furthermore, adding SAM further improved performance, possibly because this module enhances the spatial focus of high-level features and achieves more effective synergistic fusion with mid- and low-level features (Table 5). The improved model integrating all modules achieved optimal performance across all evaluation metrics: Precision 0.947, Recall 0.895, F1 0.920, mAP@0.5 0.944, and mAP@0.5–0.95 0.703 (Table 5), fully demonstrating the synergistic effect of the multi-module architecture. Despite the fact that this multi-module configuration involves more parameters, our proposed model is still more lightweight than the baseline model while significantly improving detection accuracy and robustness, achieving a good balance between accuracy and computational efficiency.

### Model visualization

To further investigate the impact of each module on the model’s attention and decision-making, and to visually validate the quantitative results of the ablation experiments, this section employs Gradient-weighted Class Activation Mapping (Grad-CAM)^54^ to generate heat maps. Grad-CAM weights the gradient information of a specific convolutional layer and maps it onto the feature map, providing an intuitive visualization of the model’s focus.

**Fig. 7:**
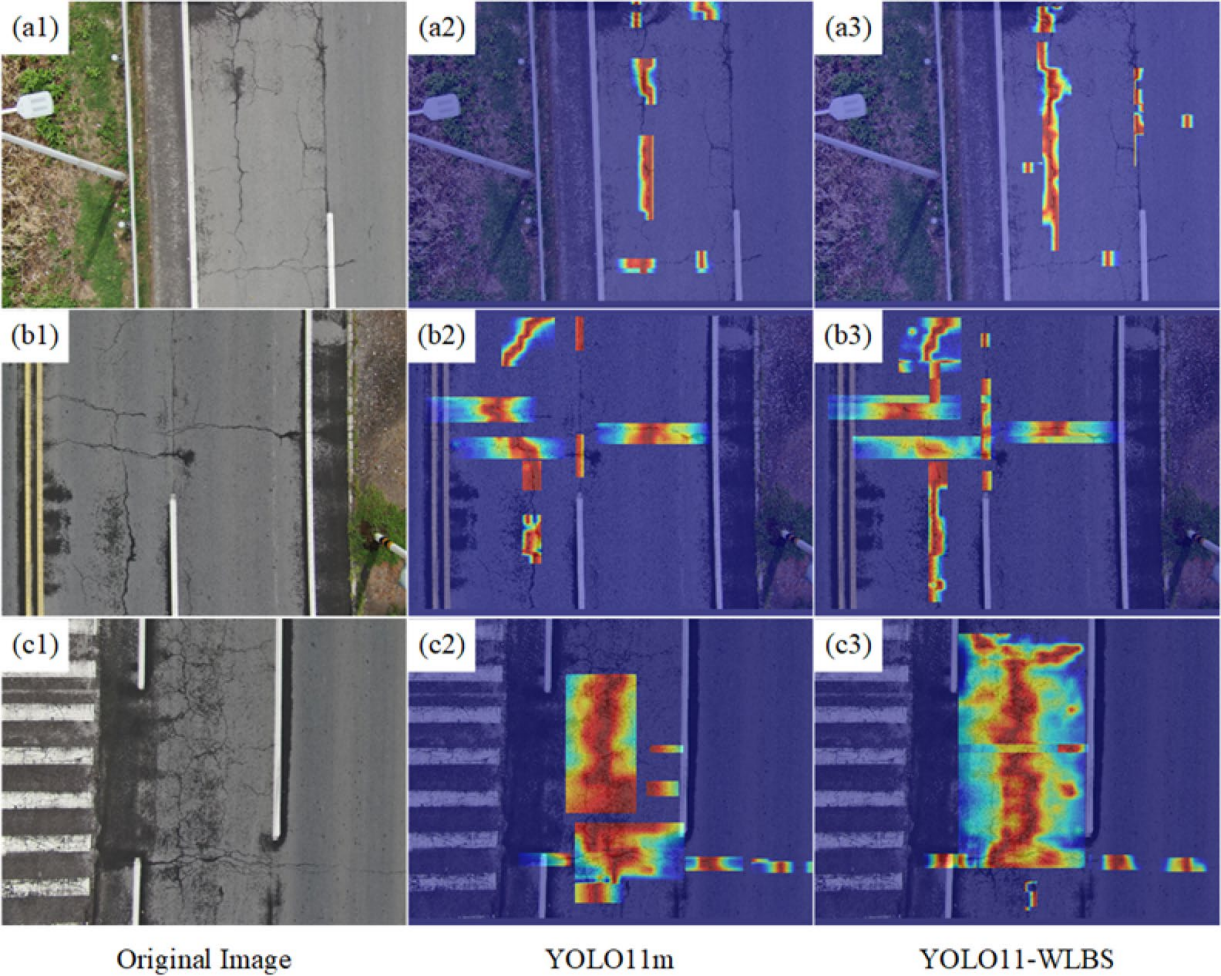
Heatmap visualization of YOLO11 and YOLO11-WLBS.

Figure 7a and b, and 7c respectively represent three typical pavement defect images from the original dataset, where the primary defect in Fig. 7a is longitudinal crack, the main defects in Fig. 7b are transverse and longitudinal cracks, and the defect in Fig. 7c is alligator cracks. Figure 7a2 ~ c2 and a3 ~ c3 respectively show the heatmap visualization results of YOLO11m and YOLO11-WBLS, which are used to compare the distribution of regions of interest.

The results show significant differences in attention distribution between the YOLO11m and the improved YOLO11-WLBS model (Fig. 7), particularly in capturing defect edges and geometric integrity. For examples, there are two main longitudinal cracks in the original image in Fig. 7a1. The YOLO11m model exhibits a relatively dispersed and fragmented attention distribution, failing to fully capture the crack’s geometric (Fig. 7a2). In contrast, the YOLO11-WLBS model demonstrates precise attention to the entire cracks, preserving the intricate edge details and continuity of the defects (Fig. 7a3). In Fig. 7b1, the defect in the original image mainly consists of a mixture of transverse and longitudinal cracks. The YOLO11m model exhibits only partial attention to defect regions (Fig. 7b2), causing nearly half of the defects area to be undetected and critical edge information to be lost, while the enhanced model of YOLO11-WLBS effectively identifies all defect regions, preserving their complete structural configuration (Fig. 7b3). Figure 7c1 shows an alligator crack. YOLO11-WLBS captures the complex network of interconnected cracks with high fidelity, preserving edge details and the complete morphology of the defect (Fig. 7c3). In contrast, YOLO11m’s attention coverage is incomplete, resulting in a large loss of key edge features and failing to fully present the overall crack pattern (Fig. 7c2).

In summary, the heatmap visualization results clearly show that our proposed model effectively preserves the edge information of pavement defects. Even when multiple defect types and multi-scale defects appear at the same time, the model can still accurately identify all types of defects. The visualization analysis clearly demonstrates the improved model’s ability to extract pavement defect edges and multi-scale features.

### Model performance under extreme conditions

**Fig. 8:**
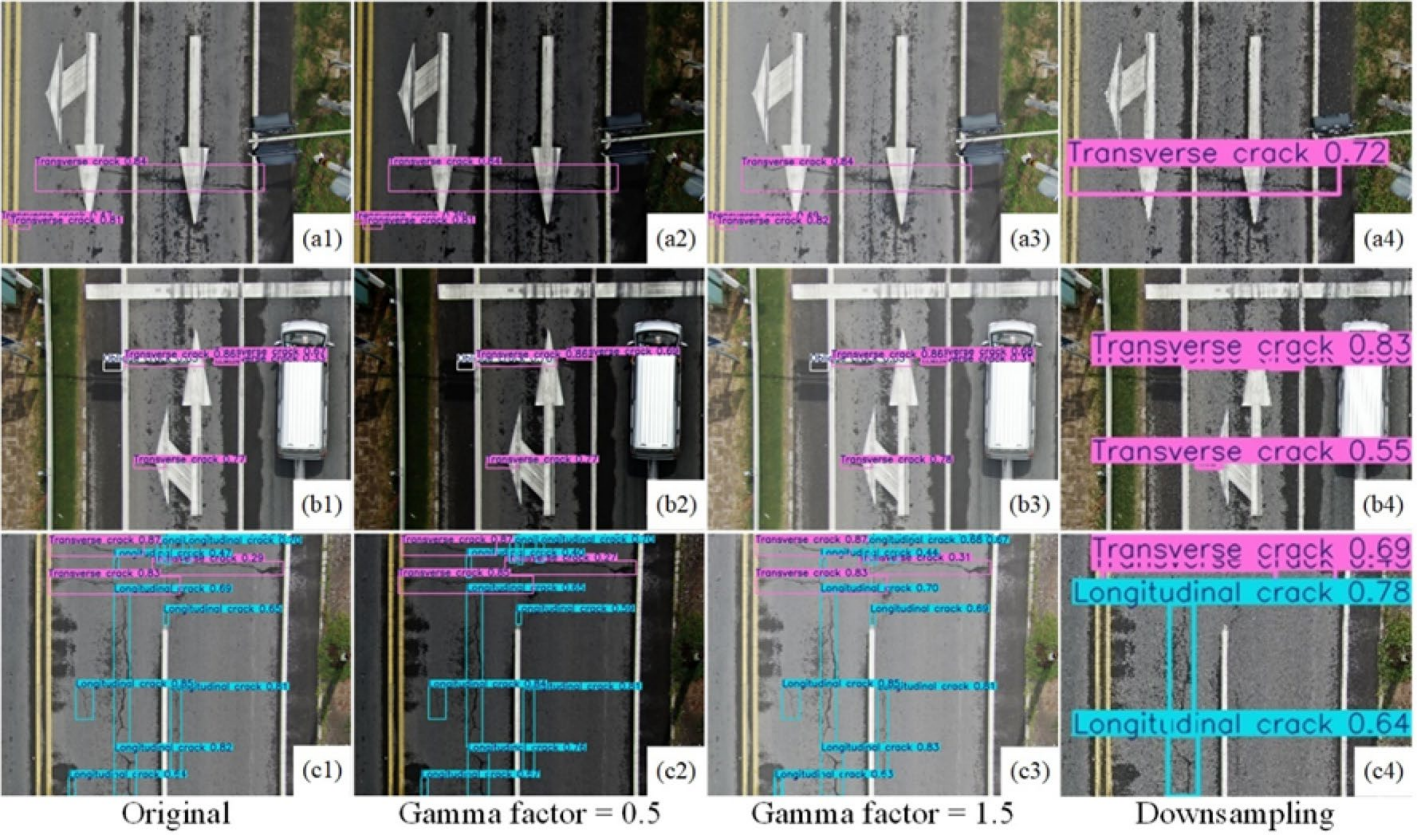
Test results under extreme conditions.

In actual engineering applications, the lighting conditions during pavement image acquisition are affected by factors such as time, weather, and pavement location. Both low-light and high-exposure environments can degrade image quality, leading to distortion of defect edges and details, complicating the detection process. In addition, low or blurred image resolution due to equipment limitations or acquisition methods may hinder the extraction of relevant defect features. To evaluate the performance of the model under extreme conditions, we use two methods: gamma correction and downsampling. gamma correction^55^ uses nonlinear transformation to adjust image brightness to simulate low-light and high-exposure environments. In this paper, the gamma factors are 0.5 and 1.5 to simulate low-light and high-light scenes, respectively. Downsampling was implemented using bilinear interpolation to simulate low-resolution and blurred imaging conditions, with the downsampling factor set to 0.1^56^. The reliability of the model under conditions of image quality degradation is evaluated by these methods. The test results are shown in Fig. 8.

Experimental results demonstrate that YOLO11-WLBS maintains stable detection performance under extreme imaging conditions, including low illumination, high illumination, and image blur (Fig. 8), demonstrating its robustness and reliability in complex visual scenes. Specifically, the three example images in Fig. 8 show that the model’s lesion detection precision under low and high illumination conditions is roughly consistent with that under good illumination, demonstrating the model’s adaptability to varying illumination conditions. Notably, the results in Fig. 8c show that, under strong illumination, the model’s detection accuracy is even slightly higher than under good illumination, further validating the improved model’s reliability in extreme illumination scenarios. Furthermore, while detection accuracy decreases somewhat by downsampling the image to simulate blur, the overall recall remains high, with no significant missed or false detections, further demonstrating the model’s robustness to image quality degradation.

### Generalization of the model

In real-world pavement detection, a model’s generalization ability is a key indicator of its adaptability and practicality^57^. Although the YOLO11-WLBS model proposed in this study demonstrates high detection accuracy on the original training dataset (Table 3), a critical concern arises: if the dataset distribution changes, or if the model encounters interference from non-training scenarios during practical application, will the model retain satisfactory accuracy? Assessing the model’s adaptability in unknown environments is therefore crucial for evaluating its practical engineering value in multi-module pavement detection systems.

Therefore, in this section, we also applied the proposed YOLO-WLBS model to the UAPD dataset^58^. This dataset contains 2401 real pavement defect images taken by drones, and the annotation system is consistent with the original training set, ensuring the comparability of performance indicators.

The results indicate that the model still maintains high detection accuracy, with a P of 0.869, a R of 0.813, a F1 of 0.840, an mAP@0.5 of 0.824, and an mAP@0.5–0.95 of 0.603. Compared with its performance on UAV-PDD2023 (Table 5), the accuracy has decreased slightly. This can probably be attributed to the reduced image resolution (the image resolution of UAPD is 500 × 500, while the image resolution of UAV-PDD2023 is 2592 × 1944) and the difference in the drone’s viewpoint. We note that although the overall performance of YOLO11-WLBS on UAPD has slightly decreased compared to the original training dataset, the precision remains above 0.85, which is sufficient to meet the engineering requirements. The new dataset validated the reliability and robustness of the YOLO11-WLBS model across diverse environments, demonstrating its strong generalization capabilities. This provides strong support for the model’s widespread adoption in practical applications such as drone inspections and mobile intelligent road monitoring, and lays a solid foundation for subsequent deployment in multiple scenarios.

## Discussion

Though our experiments have verified that the proposed model achieves high precision (reaching 0.947) in pavement defects detection, the prior tests lacked detailed classification of defect types. This limitation prevents a comprehensive reflection of the model’s adaptability to diverse pavement defects. Thus, to further verify the model’s detection performance under different defect scenarios, especially for rare defect categories, we specifically designed this test to evaluate the model’s ability to detect various common defects.

**Table 6:** Performance evaluation of different pavement defect categories.

Class	*P*	*R*	mAP@0.50	mAP@0.5–0.95
All	0.947	0.895	0.944	0.703
AC	0.972	0.962	0.992	0.812
LC	0.958	0.941	0.978	0.699
OC	0.947	0.907	0.964	0.714
TC	0.955	0.916	0.965	0.723
Potholes	0.918	0.657	0.781	0.462
Repairs	0.934	0.988	0.984	0.809

The results indicate that YOLO11-WLBS model still demonstrates excellent overall performance in detecting various pavement defects (Table 6). The model’s detection precision for cracks is higher than its overall detection precision (Table 6). This may be due to the introduction of the WTConv module, which enhances the extraction of high-frequency texture details, enabling the network to better capture crack edges and fine-grained structural patterns. Furthermore, crack samples account for as much as 95% of the dataset (Fig. 6); with sufficient sample support, the model can more fully learn the discriminative features of the cracks. Specifically, compared to the overall index, the performance metrics P, R, mAP@0.50, and mAP@0.50–0.95 for AC defects are improved by 0.025, 0.067, 0.048, and 0.109, respectively (Table 6), indicating that the improved model has stronger discriminative ability and feature representation advantages when dealing with alligator crack defects with complex texture features, enabling the model to more accurately capture their morphological structure and obtain more reliable detection results. While the performance metrics P, R, and mAP@0.50 for LC showed slight increases, mAP@0.5–0.95 decreased slightly by 0.004(Table 6). This is likely due to the elongated shape and large-scale variation of longitudinal cracks, requiring more precise matching of the predicted bounding box to the target boundary at higher IoU thresholds, leading to slight performance fluctuations.

The results also show that the detection accuracy of potholes and repairs is slightly lower than the model’s overall detection. The model achieves a detection precision of 0.918, a recall of 0.657, mAP@0.50 of 0.781, and mAP@0.50–0.95 of 0.462 for potholes. For repairs, the model yields a precision of 0.934, a recall of 0.988, mAP@0.50 of 0.984, and mAP@0.50–0.95 of 0.809. While the detection precision of potholes and repairs is slightly lower than the model’s overall detection precision, it is noteworthy that the detection precision of these specific defects still remains stably above 90%. This verifies that the proposed model in this study possesses reliable pavement defect detection capability. We note that the model achieves relatively high detection precision (0.918) but a low recall (only 0.657) for potholes. This may be attributed to the extremely limited number of pothole samples in the dataset (accounting for only 2% as illustrated in Fig. 6). The scarcity of pothole samples may constrain the model’s ability to learn their features. Furthermore, potholes are characterized by irregular boundaries and large scale variations in their appearance, and are highly susceptible to interference from environmental factors such as shadows, water accumulation, and reflections. These features significantly increase the difficulty of model recognition, making some potholes prone to misclassification or missed detection. Additionally, while the integration of WTConv enhances the model’s capacity to capture high-frequency features, thereby significantly boosting the detection performance of crack-type defects (TC, LC, AC, OC), this design may compromise the learning of low-frequency structural information, which is critical for pothole detection.

Based on the advantages demonstrated by the YOLO-WLBS model, such as high accuracy and lightweight design, here we further deploy the model on mobile devices to verify its engineering practicality. We use TensorFlow Lite^59^ as the model deployment framework and achieve the model’s adaptation to mobile platforms using full-integer (INT8) quantization^60^ (both network weights and activation values are quantized to 8-bit integers and the entire inference pipeline uses integer-only arithmetic), and layer pruning^61^. Mobile inference was performed on a Redmi K90 Pro Max smartphone equipped with 12 GB of RAM, 256 GB of storage, a 50-megapixel camera, and running Android 16, enabling its application in real-time pavement defect detection. The application uses a smartphone camera to capture real-time pavement images and can automatically identify and localize cracks, potholes, and repaired areas with high precision.

**Fig. 9:**
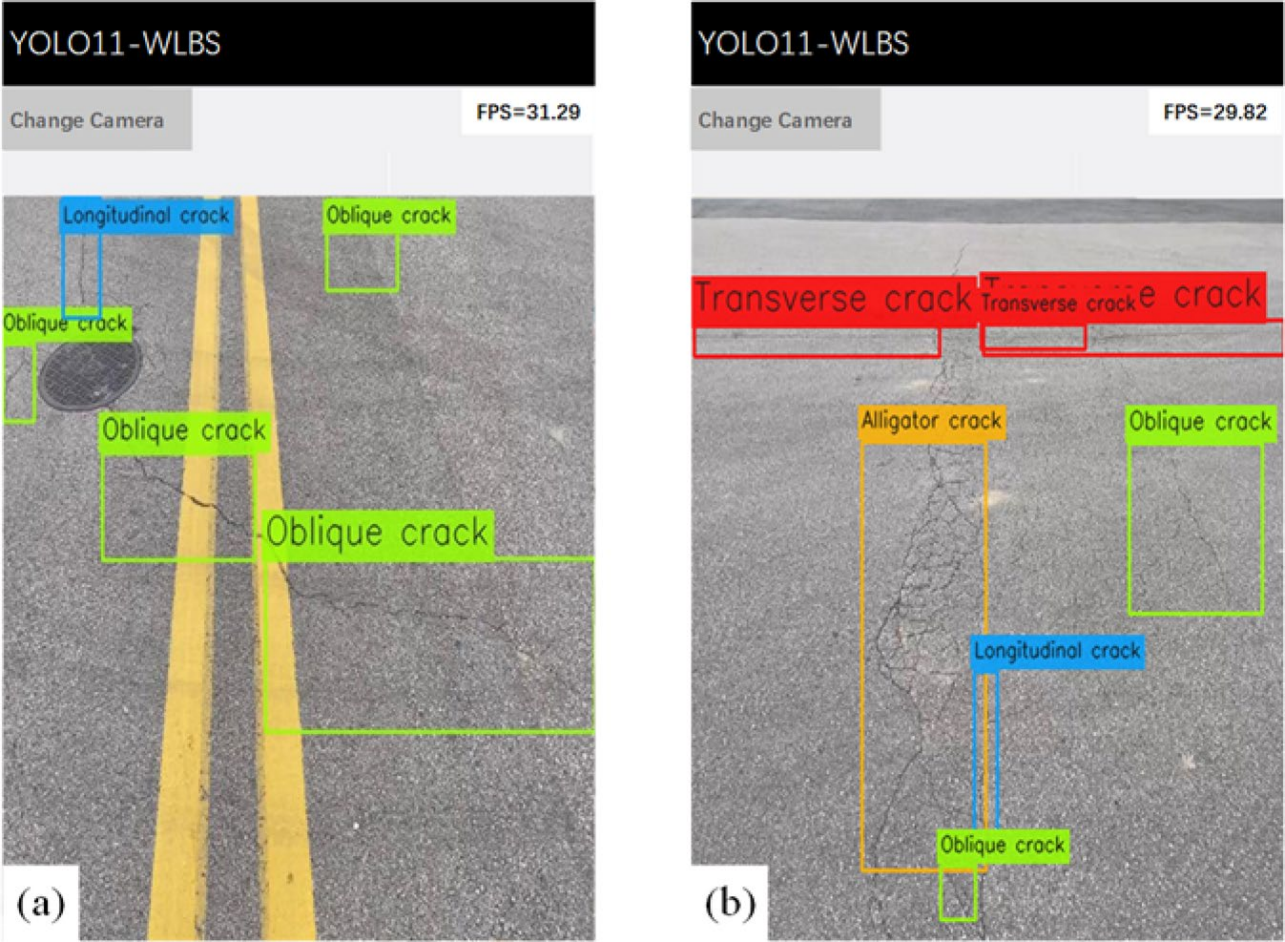
YOLO11-WLBS Android interface for real-time pavement defect.

Figure 9 shows the real-time pavement images captured on-site at an urban branch road using the smartphone camera. As observed from Fig. 9a, the pavement defects in this section are primarily oblique cracks and longitudinal cracks, with no other significant defects—this aligns with the typical defect distribution of urban branch roads. In contrast, Fig. 9b shows a more complex section of pavement defects, where oblique cracks, longitudinal cracks, transverse cracks, and alligator cracks coexist, exhibiting more severe and diverse defect patterns. The results indicate that the system can accurately capture the positions of multiple types of cracks: oblique cracks are annotated with green boxes, longitudinal cracks with blue boxes, transverse cracks with red boxes, and alligator cracks with orange boxes. The coincidence degree between the bounding boxes and the actual crack areas is relatively high. Meanwhile, the APP displays the defect types in real-time next to the annotation boxes, and the results are basically consistent with the on-site manual measurement data. All inference results are generated directly on the mobile device, without any cloud or remote acceleration. Model inference is performed on a mobile system-on-a-chip using TensorFlow Lite and employs all-integer (INT8) quantization. The frame rates reported in the upper right corner of Fig. 9 are 31.29 and 29.82, respectively, corresponding to end-to-end real-time performance. Detection results can be saved locally or uploaded for further analysis and maintenance planning. The near 30 FPS performance achieved by the proposed YOLO11-WLBS on consumer-grade mobile devices demonstrates that the model meets the real-time and efficiency requirements for edge deployment. These results (detection performance and FPS) further validate the accuracy and practicality of the proposed model in real-time detection scenarios and also confirm the effectiveness of using mobile device cameras for data acquisition.

In summary, the proposed YOLO11-WLBS model outperforms existing detectors in both accuracy and computational efficiency (Tables 3 and 5). Its strong generalization ability and lightweight design make it suitable for mobile deployment, and this has been further validated in a real-time implementation on Android devices (Fig. 9). However, there is still room for improvement in capturing low-frequency structural patterns and refining geometric feature extraction. Furthermore, this study completed the detection and classification of pavement defects (i.e., the first stage of the pavement management system). The next stage, assessing the severity of defects, is crucial for guiding subsequent maintenance prioritization. Although the current model does not directly output severity levels, its excellent performance, lightweight design, and multi-scale feature representation lay a feasible foundation for the next stage of defect severity assessment.

Future work will further introduce larger-scale, multi-source, and multi-scenario datasets, to enhance the model’s generalization ability and stability under cross-device and cross-scenario conditions. Simultaneously, it will explore multimodal information fusion strategies based on practical engineering needs to improve detection reliability and robustness under complex working conditions. It will also conduct research on multi-platform inference performance evaluation and deployment optimization, covering different hardware environments such as mobile devices, vehicle systems, and embedded devices, to improve the model’s feasibility and practical value in real-world engineering applications. At the application level, future research will also focus on the complete process of a pavement defect management system. Building upon existing defect detection and classification, it will further introduce geometric attribute modeling (e.g., crack width, length, potholes area, etc.) and a multi-task learning framework to achieve automatic quantitative assessment of defect severity, thereby supporting the scientific prioritization of maintenance and optimization of resource allocation.

## Conclusion

In order to improve the accuracy, lightweight performance and generalization ability of the pavement defect detection model, this paper proposes an enhanced model YOLO11-WLBS based on YOLO11. Through a series of systematic experiments, the following main conclusions are obtained:

(1) The introduced modules significantly improved the model’s pavement defect detection performance, confirming each module’s independent contribution. The four-module fusion achieved the best performance, showing a good synergistic enhancement effect. Among them, WTConv (high-frequency feature extraction) and BiFPN (multi-scale fusion) had the most notable effects, highlighting the key value of these two technologies in pavement defect detection.

(2) Compared with YOLO11, YOLO11-WLBS improves Precision, Recall, mAP@0.50 and mAP@0.50–0.95 by 0.064, 0.158, 0.122 and 0.058 respectively. The overall detection performance is significantly enhanced while the number of parameters is reduced by about 25.5%, which is more suitable for pavement inspection equipment (mostly embedded terminals like on-board devices) with limited computational capacity.

(3) YOLO11-WLBS maintains stable performance under complex conditions such as multi-viewpoints, multiple scenes, extreme lighting, and blurred images, showing strong generalization ability and environmental adaptability. The results indicate that the proposed model has a good engineering application potential and could meet the diverse needs of actual pavement defect detection tasks.

Despite the aforementioned advantages of YOLO11-WLBS, its integration of multiple modules and the need for network optimization result in relatively high computational resources and time required for training. Furthermore, for rare defect types (such as potholes), model performance may still be negatively impacted by severe data scarcity. Future research will focus on developing more efficient training strategies and improving datasets to further reduce training computation costs while improving detection performance for rare defects.

## Data Availability

The data used to support the findings of this study are available from the corresponding author upon reasonable request.

## References

[1] Cao, W., Liu, Q. & He, Z. Review of pavement defect detection methods. *IEEE Access.* **8**, 14531–14544. 10.1109/ACCESS.2020.2966881 (2020).

[2] Zhang, L., Xu, W., Zhu, L., Yuan, X. & Zhang, C. Study on pavement defect detection based on image processing utilizing UAV. *J. Phys: Conf. Ser.* **042011**10.1088/1742-6596/1168/4/042011 (2019).

[3] Fan, L. et al. Pavement defect detection with deep learning: A comprehensive survey. *IEEE Trans. Intell. Veh.* **9**, 4292–4311. 10.1109/TIV.2023.3326136 (2023).

[4] Zakeri, H., Nejad, F. M. & Fahimifar, A. Image based techniques for crack detection, classification and quantification in asphalt pavement: a review. *Arch. Comput. Methods Eng.* **24**, 935–977. 10.1007/s11831-016-9194-z (2017).

[5] Koch, C., Georgieva, K., Kasireddy, V., Akinci, B. & Fieguth, P. A review on computer vision based defect detection and condition assessment of concrete and asphalt civil infrastructure. *Adv. Eng. Inform.* **29**, 196–210. 10.1016/j.aei.2015.01.008 (2015).

[6] Cafiso, S., D’Agostino, C., Delfino, E. & Montella, A. From manual to automatic pavement distress detection and classification. 2017 5th IEEE International Conference on Models and Technologies for Intelligent Transportation Systems. 433–438. (2017). 10.1109/MTITS.2017.8005711

[7] Benmhahe, B. & Chentoufi, J. A. Automated pavement distress detection, classification and measurement: A review. *Int. J. Adv. Comput. Sci. Appl.* **12**10.14569/IJACSA.2021.0120882 (2021).

[8] Alnaqbi, A., Al-Khateeb, G. G. & Zeiada, W. Optimized prediction of longitudinal cracking in concrete pavements using hybrid GA-GBM models. *J. Building Pathol. Rehabilitation*. **10**, 156. 10.1007/s41024-025-00667-9 (2025).

[9] Alnaqbi, A., Al-Khateeb, G. G., Zeiada, W. & Abuzwidah, M. Random forest-based frame work for multi-distress prediction in CRCP: a feature importance approach. *Discover Civil Eng.* **2**, 140. 10.1007/s44290-025-00302-z (2025).

[10] Alnaqbi, A., Al-Khateeb, G. G. & Zeiada, W. Genetic Algorithm-Enhanced gradient boosting for transverse cracking in CRCP. *Jordan J. Civil Eng.* **19**10.14525/JJCE.v19i2.11 (2025).

[11] Ma, J., Jiang, X., Fan, A., Jiang, J. & Yan, J. Image matching from handcrafted to deep features: A survey. *Int. J. Comput. Vision*. **129**, 23–79. 10.1007/s11263-020-01359-2 (2021).

[12] Dargan, S., Kumar, M., Ayyagari, M. R. & Kumar, G. A survey of deep learning and its applications: a new paradigm to machine learning. *Arch. Comput. Methods Eng.* **27**, 1071–1092. 10.1007/s11831-019-09344-w (2020).

[13] Arya, D. et al. Deep learning-based road damage detection and classification for multiple countries. *Autom. Constr.* **132**, 103935. 10.1016/j.autcon.2021.103935 (2021).

[14] Hsieh, C. C., Jia, H. W., Huang, W. H. & Hsih, M. H. Deep Learning-Based Road Pavement Inspection by Integrating Visual Information and IMU. *Information,* **15**, 239. (2024). 10.3390/info15040239

[15] Alzubaidi, L. et al. Review of deep learning: concepts, CNN architectures, challenges, applications, future directions. *J. Big Data*. **8**, 53. 10.1186/s40537-021-00444-8 (2021). 33816053 10.1186/s40537-021-00444-8 PMC8010506

[16] Tong, Z., Gao, J. & Zhang, H. Innovative method for recognizing subgrade defects based on a convolutional neural network. *Constr. Build. Mater.* **169**, 69–82. 10.1016/j.conbuildmat.2018.02.081 (2018).

[17] Redmon, J., Divvala, S., Girshick, R. & Farhadi, A. You only look once: Unified, real-time object detection. Proceedings of the IEEE conference on computer vision and pattern recognition. 779–788 (2016).

[18] Girshick, R. & Fast, R-C-N-N. Proceedings of the IEEE international conference on computer vision. 1440–1448 (2015).

[19] Bharati, P. & Pramanik, A. Deep learning techniques—R-CNN to mask R-CNN: a survey. *Comput. Intell. Pattern Recognition: Proc. CIPR 2019*. **657–668**10.1007/978-981-13-9042-5_56 (2019).

[20] Liu, W. et al. Ssd: Single shot multibox detector. Computer Vision–ECCV. : 14th European Conference, Amsterdam, The Netherlands, October 11–14, 2016, Proceedings, Part I 14. 21–37. (2016). 10.1007/978-3-319-46448-0_2 (2016).

[21] Terven, J., Córdova-Esparza, D. M. & Romero-González J.-A. A comprehensive review of Yolo architectures in computer vision: from Yolov1 to Yolov8 and Yolo-nas. *Mach. Learn. Knowl. Extr.* **5**, 1680–1716. 10.3390/make5040083 (2023).

[22] Du, F. J. & Jiao, S. J. Improvement of lightweight convolutional neural network model based on YOLO algorithm and its research in pavement defect detection. *Sensors* **22**, 3537. 10.3390/s22093537 (2022). 35591227 10.3390/s22093537 PMC9103593

[23] Majidifard, H., Jin, P., Adu-Gyamfi, Y. & Buttlar, W. G. Pavement image datasets: A new benchmark dataset to classify and densify pavement distresses. *Transp. Res. Rec.* **2674**, 328–339. 10.1177/0361198120907283 (2020).

[24] Ma, L. & Chen, M. Road damage detection based on improved YOLO algorithm. *Sci. Rep.* **15**, 28506. 10.1038/s41598-025-14461-7 (2025). 40764422 10.1038/s41598-025-14461-7 PMC12325952

[25] Zhang, S., Bei, Z., Ling, T., Chen, Q. & Zhang, L. Research on high-precision recognition model for multi-scene asphalt pavement distresses based on deep learning. *Sci. Rep.* **14**, 25416. 10.1038/s41598-024-77173-4 (2024). 39455708 10.1038/s41598-024-77173-4 PMC11512018

[26] Li, Z. et al. Lightweight pavement crack detection model for edge computing devices. *Sci. Rep.* **15**, 38179. 10.1038/s41598-025-22092-1 (2025). 41173964 10.1038/s41598-025-22092-1 PMC12578820

[27] Dong, S. et al. Advanced lightweight deep learning vision framework for efficient pavement damage identification. *Sci. Rep.* **15**, 12966. 10.1038/s41598-025-97132-x (2025). 40234635 10.1038/s41598-025-97132-x PMC12000367

[28] Mulyanto, A., Sari, R. F., Muis, A. & Harwahyu, R. Vision-Based automated pavement distress inspection: A review. *IEEE Access.*10.1109/TITS.2021.3113802 (2025).

[29] Wang, C. et al. -HR: implementing lightweight and efficient pavement distress detection by enhancement of the Spatial information extractions of high-resolution features. *Eng. Res. Express*. **7**, 045226. 10.1088/2631-8695/ae0f01 (2025).

[30] Sapkota, R. et al. YOLO advances to its genesis: a decadal and comprehensive review of the you only look once (YOLO) series. *Artif. Intell. Rev.* **58**, 274. 10.1007/s10462-025-11253-3 (2025).

[31] Ali, M. L. & Zhang, Z. The YOLO framework: A comprehensive review of evolution, applications, and benchmarks in object detection. *Computers* **13**, 336. 10.3390/computers13120336 (2024).

[32] Jiang, P., Ergu, D., Liu, F., Cai, Y. & Ma, B. A review of Yolo algorithm developments. *Procedia Comput. Sci.* **199**, 1066–1073. 10.1016/j.procs.2022.01.135 (2022).

[33] Aicha, M. Techniques and applications of image and signal processing: A theoretical approach. *8th Int. Conf. Image Signal. Process. Their Appl. (ISPA)*. **1-8**10.1007/s10462-025-11253-3 (2024). (2024).

[34] Xiang, S. & Liang, Q. Remote sensing image compression based on high-frequency and low-frequency components. *IEEE Trans. Geosci. Remote Sens.* **62**, 1–15. 10.1109/TGRS.2023.3349306 (2024).

[35] Zhang, X., Wang, X. & Yang, Z. A. Lightweight pavement defect detection algorithm integrating perception enhancement and feature optimization. *Sensors* **25**, 4443. 10.3390/s25144443 (2025). 40732571 10.3390/s25144443 PMC12298082

[36] Daubechies, I. Orthonormal bases of compactly supported wavelets. *Commun. Pure Appl. Math.* **41**, 909–996. 10.1002/cpa.3160410705 (1988).

[37] Heil, C. E. & Walnut, D. F. Continuous and discrete wavelet transforms. *SIAM Rev.* **31**, 628–666. 10.1137/1031129 (1989).

[38] Lin, T. Y. et al. Feature pyramid networks for object detection. Proceedings of the IEEE conference on computer vision and pattern recognition. 2117–2125 (2017).

[39] Chen, P. Y., Chang, M. C., Hsieh, J. W. & Chen, Y. S. Parallel residual bi-fusion feature pyramid network for accurate single-shot object detection. *IEEE Trans. Image Process.* **30**, 9099–9111. 10.1109/TIP.2021.3118953 (2021). 34735334 10.1109/TIP.2021.3118953

[40] Zhang, H. et al. A recursive attention-enhanced bidirectional feature pyramid network for small object detection. *Multimedia Tools Appl.* **82**, 13999–14018. 10.1007/s11042-022-13951-4 (2023).

[41] Yang, L., Zhang, R. Y., Li, L., Xie, X. & Simam A simple, parameter-free attention module for convolutional neural networks. International conference on machine learning. 11863–11874 (2021).

[42] Muraina, I. Ideal dataset splitting ratios in machine learning algorithms: general concerns for data scientists and data analysts. 7th international Mardin Artuklu scientific research conference. 496–504 (2022).

[43] Padilla, R., Netto, S. L. & Da Silva, E. A. A survey on performance metrics for object-detection algorithms. 2020 international conference on systems, signals and image processing (IWSSIP). 237–242. (2020). 10.1109/IWSSIP48289.2020.9145130

[44] Arman, M. S. et al. Detection and classification of road damage using R-CNN and faster R-CNN: a deep learning approach. Cyber Security and Computer Science: Second EAI International Conference, ICONCS 2020, Dhaka, Bangladesh, February 15–16, 2020, Proceedings 2. 730–741. (2020). 10.1007/978-3-030-52856-0_58

[45] Jocher, G. et al. ultralytics/yolov5: v3. 0. *Zenodo* (2020).

[46] Sohan, M., Sai Ram, T. & Rami Reddy, C. V. A review on yolov8 and its advancements. International Conference on Data Intelligence and Cognitive Informatics. 529–545. (2024). 10.1007/978-981-99-7962-2_39

[47] Wang, C. Y., Yeh, I. H. & Mark Liao, H. Y. Yolov9: Learning what you want to learn using programmable gradient information. European conference on computer vision. 1–21. (2024). 10.1007/978-3-031-72751-1_1

[48] Wang, A. et al. Yolov10: Real-time end-to-end object detection. *Adv. Neural. Inf. Process. Syst.* **37**, 107984–108011. 10.52202/079017-3429 (2024).

[49] Khanam, R. & Hussain, M. Yolov11: an overview of the key architectural enhancements. *ArXiv Preprint arXiv:2410 17725*. 10.48550/arXiv.2410.17725 (2024).

[50] Insany, G. P., Indriyani, R., Ma’wa, N. J. & Safitri, S. Performance analysis of YOLOv11: Nano, Small, and medium models for herbal leaf classification. *Eng. Proc.* **107**, 102. 10.3390/engproc2025107102 (2025).

[51] He, L., Zhou, Y., Liu, L., Cao, W. & Ma, J. -h. Research on object detection and recognition in remote sensing images based on YOLOv11. *Sci. Rep.* **15**, 14032. 10.1038/s41598-025-96314-x (2025). 40269047 10.1038/s41598-025-96314-x PMC12019343

[52] Jegham, N., Koh, C. Y., Abdelatti, M. & Hendawi, A. Yolo evolution: A comprehensive benchmark and architectural review of yolov12, yolo11, and their previous versions. *arXiv preprint arXiv:2411.00201*. (2024). 10.48550/arXiv.2411.00201

[53] Zeiler, M. D. & Fergus, R. Visualizing and understanding convolutional networks. European conference on computer vision. 818–833. (2014). 10.1007/978-3-319-10590-1_53

[54] Selvaraju, R. R. et al. Grad-cam: Visual explanations from deep networks via gradient-based localization. Proceedings of the IEEE international conference on computer vision. 618–626 (2017).

[55] Rahman, S., Rahman, M. M., Abdullah-Al-Wadud, M., Al-Quaderi, G. D. & Shoyaib, M. An adaptive gamma correction for image enhancement. *EURASIP J. Image Video Process.* **2016, **35. 10.1186/s13640-016-0138-1 (2016).

[56] Dumitrescu, D. & Boiangiu, C. A. A study of image upsampling and downsampling filters. *Computers* **8**, 30. 10.3390/computers8020030 (2019).

[57] Packer, C. et al. Assessing generalization in deep reinforcement learning. *ArXiv Preprint arXiv:1810 12282*. 10.48550/arXiv.1810.12282 (2018).

[58] Zhang, Y. et al. Road damage detection using UAV images based on multi-level attention mechanism. *Autom. Constr.* **144**, 104613. 10.1016/j.autcon.2022.104613 (2022).

[59] David, R. et al. Tensorflow lite micro: Embedded machine learning for tinyml systems. *Proc. Mach. Learn. Res.* **3**, 800–811 (2021).

[60] Kim, S., Park, G. & Yi, Y. Performance evaluation of INT8 quantized inference on mobile GPUs. *IEEE Access.* **9**, 164245–164255. 10.1109/ACCESS.2021.3133100 (2021).

[61] Sietsma & Dow. international conference on neural networks. 325–333 vol. 321. Neural net pruning-why and how. IEEE (1988). 10.1109/ICNN.1988.23864 (1988).

